# Meteorological Register

**Published:** 1814-08

**Authors:** C. Blunt

**Affiliations:** Philosophical Instrument Maker, No. 38, Tavistock-Street, Covent-Garden


### METEOROLOGICAL REGISTER.

*From June the 25th, to July the 25th,* 1814.

Kept by C. BLUNT, Philosophical Instrument Maker, No. 38, Tavistock-Street, Covent-Garden.

| Moon. | Day. | Wind. | Barometrical Pressure. Max. | Min. | Mean. | Temperature. Max. | Min. | Mean. | |
|---|---|---|---|---|---|---|---|---|---|
| | 26 | NW | 30·08 | 30·08 | 30·08 | 59 | 48 | 53·6 | Cloudy Small Rain |
| | 27 | N | 30·05 | 30·— | 30·03 | 61 | 49 | 54·6 | Clear |
| | 28 | NW | 29·98 | 29·93 | 29·945 | 68 | 50 | 58·6 | Fair |
| | 29 | NW | 29·91 | 29·89 | 29 897 | 73 | 51 | 61·3 | Fair |
| | 30 | NW | 29·94 | 29·92 | 29·932 | 71 | 53 | 62 | Fair |
| | 1 | NW | 29·90 | 29·88 | 29·885 | 72 | 51 | 61·3 | Fair |
| ○ | 2 | NW | 30 01 | 29·94 | 29·992 | 72 | 52 | 62 | Fair |
| | 3 | NW | 30·13 | 30·— | 30·042 | 73 | 56 | 64·3 | Fair |
| | 4 | NW | 30·05 | 30·04 | 30·042 | 74 | 57 | 64·6 | Fair, Rain during the Night |
| | 5 | NW | 30·05 | 30·03 | 30·04 | 73 | 58 | 64·3 | Fair |
| | 6 | NW | 30 05 | 30·04 | 30·045 | 73 | 59 | 64·3 | Fair |
| | 7 | NW | 30 04 | 29·93 | 29·975 | 74 | 57 | 63 6 | Rainy |
| | 8 | W | 29·93 | 29 83 | 29·82 | 75 | 61 | 65 | Rainy |
| | 9 | W | 29·83 | 29 74 | 29 827 | 74 | 61 | 67 | Rainy |
| ◑ | 10 | W | 29 73 | 29·68 | 29·705 | 73 | 58 | 65 | Showery |
| | 11 | W | 29·93 | 29 90 | 29·907 | 72 | 58 | 63·6 | Rainy, very heavy showers |
| | 12 | NW | 30·06 | 30·01 | 30·047 | 73 | 56 | 63 6 | Fr |
| | 13 | NW | 30·07 | 29·95 | 30·— | 70 | 56 | 62·3 | Fr |
| | 14 | NW | 29 83 | 29 76 | 29 935 | 71 | 57 | 63·6 | Fr |
| | 15 | W | 29 76 | 29·73 | 29·987 | 70 | 56 | 62 | Fair |
| | 16 | N | 29 80 | 29·64 | 29·982 | 72 | 58 | 63·3 | Fair |
| ● | 17 | SW | 29·86 | 29·84 | 29·85 | 71 | 55 | 62·3 | Fair |
| | 18 | W | 29·89 | 29·86 | 29 882 | 69 | 54 | 61 | Fair |
| | 19 | NW | 29 88 | 29·83 | 29·857 | 70 | 56 | 62 | Fair |
| | 20 | W | 29·70 | 29 67 | 29·685 | 71 | 55 | 62 6 | Fair |
| | 21 | SW | 29·84 | 29·73 | 29·802 | 74 | 56 | 64·3 | Fair |
| | 22 | W | 30·08 | 29·87 | 29·995 | 75 | 57 | 65·3 | Fair |
| | 23 | SW | 30·20 | 30·18 | 30·195 | 76 | 57 | 66·3 | Fair |
| ◐ | 24 | SW | 30·14 | 30·— | 30 07 | 79 | 58 | 67·6 | Fair |
| | 25 | SW | 29 78 | 29·74 | 29 26 | 83 | 58 | 69·6 | Fair |

RESULTS.

| | | | | | |
|---|---|---|---|---|---|
| Mean barometrical pressure 29·9237 | | | Mean temperature | | 63·03 deg. |
| Maximum 30·20 | wind at | SW | Maximum 83, | the wind at | SW |
| Minimum 29 64 | ——— | N | Minimum 48, | ——— | NW |

*Scale exhibiting the prevailing Winds during the Month.*

| N | NE | E | SE | S | SW | W | NW |
|---|---|---|---|---|---|---|---|
| 2 | 0 | 0 | 0 | 0 | 5 | 8 | 15 |

| | Mean barometrical pressure. | Mean temperature. |
|---|---|---|
| From the full moon on the 2d July, to the last quarter on the 10th | 29·96 | 65·83 |
| ——— last quarter, to the new moon on the 17th | 29·937 | 63·34 |
| ——— new moon, to the first quarter on the 24th | 29·899 | 63·— |

3 Measles,

Measles, hooping-cough, scarlatina anginosa, and small-pox, are the prevailing epidemics of the season. Some cases of synochus have occurred, and diarrhœa and bilious affections are not uncommon. At the Asylum for Female Orphans, some cases of small-pox after vaccination have very recently appeared, but of a very mild sort, and attended with little indisposition, though in some of the children there was a pretty good crop of pustules.

